# Risk of osteoporosis and fracture among patients with breast cancer: a systematic review and meta-analysis of cohort studies

**DOI:** 10.3389/fonc.2026.1905334

**Published:** 2026-08-31

**Authors:** Ke Li, Yanan Qian, Yujing Cao

**Affiliations:** Faculty of Traumatology and Orthopedics, Henan University of Chinese Medicine, Zhengzhou, Henan, China

**Keywords:** aromatase inhibitor, breast cancer, fracture, meta-analysis, osteoporosis

## Abstract

**Background:**

Breast cancer survivors may be at increased risk of osteoporosis and fractures because of disease-related factors and treatment-induced bone loss. However, the available evidence remains inconsistent. This study aimed to evaluate the associations between breast cancer and the risks of osteoporosis and fracture through a systematic review and meta-analysis.

**Methods:**

PubMed, Embase, Web of Science, and the Cochrane Library were systematically searched from database inception to May 1, 2026. Cohort studies investigating the associations between breast cancer and the risks of osteoporosis or fracture were included. Hazard ratios (HRs) and corresponding 95% confidence intervals (CIs) were pooled using random-effects models. Subgroup analyses were performed according to age and treatment modality.

**Results:**

A total of 16 cohort studies were included in this meta-analysis. The pooled analysis demonstrated that breast cancer was associated with significantly increased risks of osteoporosis (HR = 1.54, 95% CI: 1.13-2.09) and fracture (HR = 1.49, 95% CI: 1.03-2.16). Age-stratified findings suggested comparable osteoporosis risks between women aged <50 years and those aged ≥50 years. In the treatment subgroup analysis, aromatase inhibitor therapy was associated with a significantly increased risk of osteoporosis (HR = 1.65, 95% CI: 1.05-2.60), whereas no significant associations were observed among tamoxifen users or patients receiving chemotherapy.

**Conclusion:**

Breast cancer is significantly associated with increased risks of both osteoporosis and fracture. Aromatase inhibitor therapy may further contribute to osteoporosis risk. These findings highlight the importance of bone health monitoring and fracture prevention strategies in breast cancer survivors.

**Systematic Review Registration:**

https://www.crd.york.ac.uk/PROSPERO/view/CRD420261419796, identifier CRD420261419796.

## Introduction

Breast cancer is the most commonly diagnosed malignancy among women worldwide and remains a major public health challenge (1–3). Advances in screening, diagnosis, and treatment have substantially improved survival outcomes, resulting in a rapidly growing population of breast cancer survivors (4–6). Consequently, increasing attention has been directed toward the long-term complications associated with breast cancer and its treatment.

Osteoporosis is a systemic skeletal disorder characterized by low bone mass and deterioration of bone microarchitecture, leading to increased bone fragility and fracture risk (7, 8). Osteoporotic fractures are associated with substantial morbidity, mortality, and healthcare costs worldwide (9, 10). Identifying populations at high risk for osteoporosis and fractures is therefore of considerable clinical importance.

Accumulating evidence suggests that breast cancer patients may be at increased risk of osteoporosis and fractures (11–13). Several mechanisms have been proposed, including estrogen deficiency induced by endocrine therapy, chemotherapy-related ovarian failure, premature menopause, reduced physical activity, and nutritional deficiencies. In particular, aromatase inhibitors have been associated with accelerated bone loss because of their profound estrogen-lowering effects (14, 15). However, the relationship between breast cancer and the risks of osteoporosis and fracture, considering both disease-related factors and treatment-induced bone loss, remains incompletely understood. Furthermore, the potential influences of age and treatment modality on skeletal outcomes remain unclear (16).

To provide a more comprehensive evaluation of the available evidence, we conducted a systematic review and meta-analysis to investigate the associations between breast cancer and the risks of osteoporosis and fracture. In addition, subgroup analyses according to age and treatment modality were performed to explore potential sources of heterogeneity and provide evidence for bone health management in breast cancer survivors.

## Methods

This systematic review and meta-analysis was carried out following the Preferred Reporting Items for Systematic Reviews and Meta-Analyses (PRISMA) guidelines (17). The study protocol was registered on PROSPERO (CRD420261419796).

### Search strategy

A comprehensive literature search was conducted in PubMed, Embase, Web of Science, and the Cochrane Library to identify eligible cohort studies published up to May 1, 2026. The search strategy combined terms related to breast cancer and skeletal outcomes, including “breast cancer”, “breast neoplasm”, “osteoporosis”, “bone loss”, and “fracture”. Detailed search strategies for each database are provided in **Supplementary File**. In addition, the reference lists of all eligible articles and relevant reviews were manually screened to identify potentially missed studies. Only studies published in English were considered eligible.

### Eligibility criteria

To evaluate the longitudinal association between breast cancer and subsequent skeletal outcomes, only cohort studies were included in this systematic review and meta-analysis. Cohort studies allow assessment of the temporal relationship between breast cancer diagnosis and the occurrence of osteoporosis and fracture, and provide reliable estimates of long-term risks in real-world clinical populations.

Studies were considered eligible if they met the following criteria: (1) Participants were adults with a diagnosis of breast cancer. Studies were not restricted by molecular subtype, clinical stage, or treatment modality, and all eligible breast cancer populations were considered for inclusion. Studies were excluded only when the characteristics of the breast cancer population or skeletal outcomes could not be clearly identified; (2) the study included a comparison group without breast cancer or reported standardized risk estimates relative to the general population; (3) the outcomes of interest were osteoporosis and/or fracture. Osteoporosis was defined according to the diagnostic criteria used in each included study, including clinical diagnosis, bone mineral density assessment, or validated administrative database codes. Fracture outcomes included clinically diagnosed or registry-recorded fractures as reported by the original studies, without restriction to specific anatomical sites; (4) adjusted effect estimates, including hazard ratios (HRs), together with their corresponding 95% confidence intervals (CIs), were reported or could be calculated; and (5) the study adopted a cohort design.

Studies were excluded if they met any of the following criteria: (1) case reports, case series, cross-sectional studies, case-control studies, reviews, meta-analyses, editorials, guidelines, or conference abstracts; (2) insufficient data for effect size estimation; (3) duplicate publications derived from the same study population; or (4) the full text was unavailable.

### Study selection

**Figure 1:**
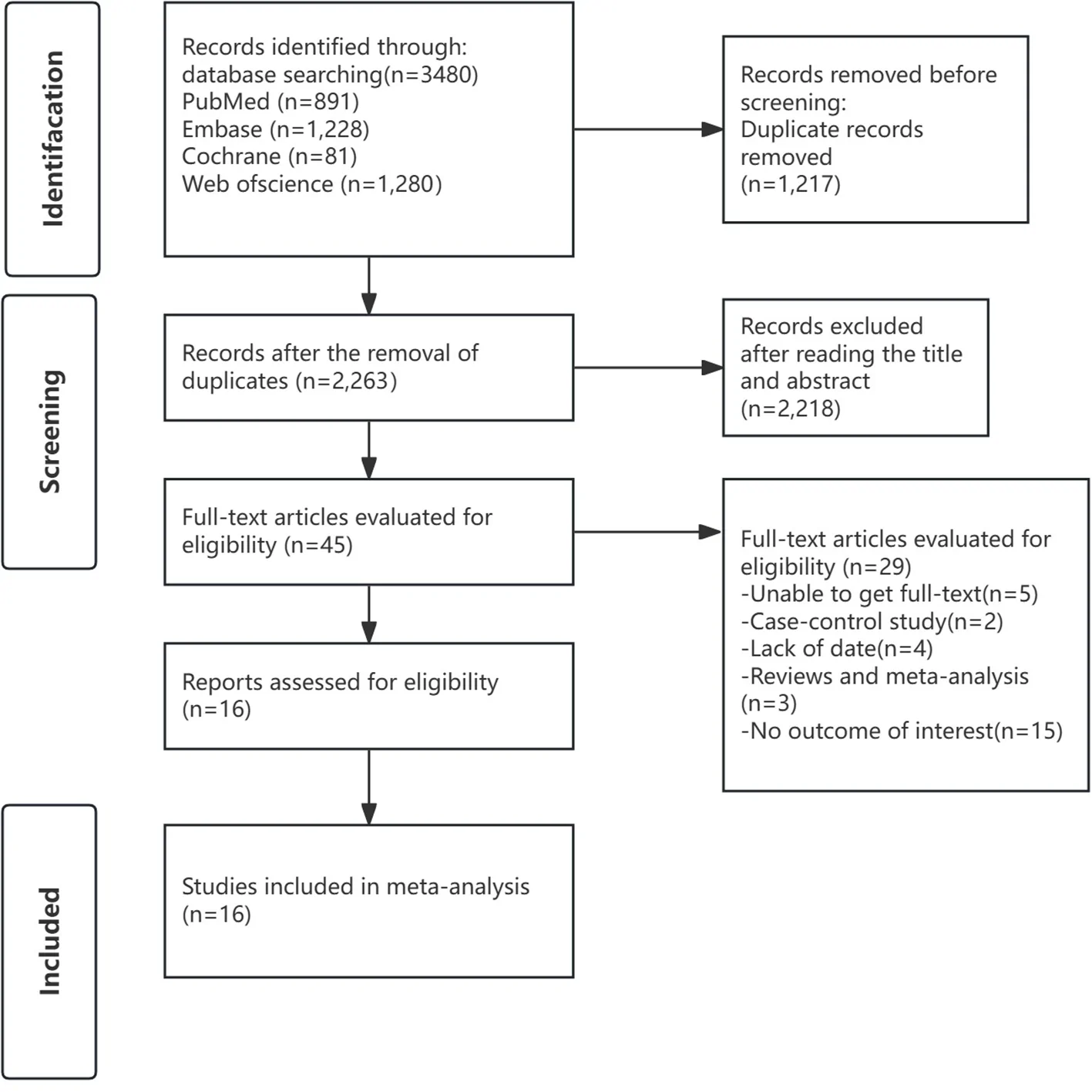
Search strategy diagram.

Two reviewers independently screened the titles and abstracts of all retrieved records. After removing duplicates and clearly irrelevant publications, the full texts of potentially eligible studies were assessed for inclusion. Any disagreement regarding study eligibility was resolved through discussion, and when necessary, consultation with a third reviewer was sought to achieve consensus. The study selection process is illustrated in the PRISMA flow diagram (**Figure 1**).

### Data extraction

Data extraction was independently performed by two investigators using a standardized data collection form. The following information was obtained from each study: author and year of publication, study design, country, study period, sample size, age, follow-up time, outcome, effect estimates (HRs, RRs, or SIRs), corresponding 95% confidence intervals, and NOS score. Any discrepancies in data extraction were resolved through discussion and consensus.

### Risk of bias assessment

The methodological quality of the included cohort studies was evaluated using the Newcastle-Ottawa Scale (NOS). This instrument assesses three domains: selection of study groups, comparability of cohorts, and outcome assessment. A maximum of four stars can be awarded for selection, two for comparability, and three for outcome assessment, yielding a total score ranging from 0 to 9. Two reviewers independently conducted the quality assessment, and disagreements were resolved through discussion.

### Statistical analysis

Adjusted effect estimates and their corresponding 95% CIs were extracted from each study and pooled to evaluate the association between breast cancer and the risk of osteoporosis and fracture. Hazard ratios (HRs), relative risks (RRs), and standardized incidence ratios (SIRs) were extracted from the included studies. Although these measures represent different statistical quantities, they were considered to provide comparable estimates of the relative association between breast cancer and skeletal outcomes, given the relatively uncommon occurrence of osteoporosis and fracture outcomes and the observational nature of the included studies. Potential methodological heterogeneity arising from differences in effect measures was acknowledged as a limitation and was considered when interpreting the pooled estimates.

Statistical heterogeneity across studies was assessed using Cochran’s Q test and the I² statistic. Considering the expected clinical and methodological heterogeneity among the included cohorts, including differences in geographic regions, patient characteristics, treatment regimens, follow-up durations, and outcome definitions, random-effects models were applied for all pooled analyses. The between-study variance was estimated using the restricted maximum-likelihood (REML) method, and the corresponding 95% confidence intervals were calculated accordingly. This approach assumes that the true effect sizes may vary across studies and provides more conservative estimates when between-study variability exists.

Subgroup analyses were conducted according to age, treatment modality (tamoxifen, aromatase inhibitor, or chemotherapy), and outcome type (osteoporosis or fracture), where sufficient data were available from the included studies. When relevant information was unavailable, we attempted to contact the corresponding study authors to obtain additional data. Publication bias was assessed through visual inspection of funnel plots and Begg’s rank correlation test. Given the limited number of included studies, the results of publication bias assessments were interpreted cautiously, as both funnel plot asymmetry and statistical tests may have limited power when the number of studies is small. All statistical analyses were performed using R software (version 4.4.1), with the meta package used for conducting the meta-analyses.

## Results

### Literature search

The literature search identified 3,480 potentially relevant records published before May 1, 2026. After duplicate removal, 1,217 articles remained for title and abstract screening. Following the initial screening process, 1,172 records were excluded because they did not meet the eligibility criteria. The full texts of 45 potentially eligible studies were subsequently reviewed in detail, resulting in the exclusion of 29 articles. The reasons for exclusion are presented in **Supplementary File**. Ultimately, 16 cohort studies were included in the final meta-analysis. The study selection process is illustrated in **Figure 1**.

### Study characteristics

The main characteristics of the 16 included cohort studies (11–13, 16, 18–29) are summarized in **Table 1**. All included studies were cohort studies published between 2011 and 2026, involving a total of 1,480,543 participants. The study populations were drawn from several countries, including South Korea, the United Kingdom, China, Japan, Israel, the United States, and Canada. Among the included studies, six were conducted in South Korea (11, 19, 22–25), four in the United Kingdom (12, 13, 16, 21), two in Israel (18, 29), and one each in China, Japan, the United States, and Canada. The study periods ranged from 1998 to 2020. The mean age of participants ranged from 43 to 69 years. Regarding study outcomes, eleven studies evaluated osteoporosis risk (18–25, 27–29), three assessed fracture risk (11, 12, 16), and two reported both osteoporosis and fracture outcomes (13, 26). The follow-up duration ranged from 1 to 10 years.

**Table 1:** Basic characteristics of included studies.

Author(year)	Research type	Country	Study period	Sample	Age	follow-up time (years)	Outcome	NOS scores
Kwak et al. (2025) (23)	Cohort	South Korea	2008-2016	63,021	49 means	8	Osteoporosis	9
Lee et al. (2025) (25)	Cohort	South Korea	2009-2015	80,661	60 means	9	Osteoporosis	8
Kang et al. (2022) (19)	Cohort	South Korea	2002-2016	84,969	46 means	5	Osteoporosis	8
Khan et al. (2011) (21)	Cohort	UK	2003-2006	26,213	67 means	6	Osteoporosis	7
Liu et al. (2025) (27)	Cohort	China	2003-2018	3,703	60 means	5	Osteoporosis	6
Kawamura et al. (2025) (20)	Cohort	Japan	2003-2008	120,085	50.5 means	3	Osteoporosis	8
Buzasi et al. (2024) (13)	Cohort	UK	1998-2020	578,160	65 means	4	Osteoporosis, Fracture	8
Kim et al. (2024) (22)	Cohort	South Korea	2009-2015	4,654	50 means	7	Osteoporosis	6
Rouach et al. (2023) (29)	Cohort	Israel	2009-2020	8,617	62.8 means	6	Osteoporosis	6
Lee et al. (2020) (24)	Cohort	South Korea	2007-2017	47,649	49 means	1	Osteoporosis	7
Ramin et al. (2018) (28)	Cohort	USA	2005-2013	778	47 means	6	Osteoporosis	6
Hamood et al. (2019) (18)	Cohort	Israel	2002-2012	1,692	49 means	5	Osteoporosis	6
Stumpf et al. (2019) (12)	Cohort	UK	2005-2015	3,522	43 means	5	Fracture	6
Cho et al. (2026) (11)	Cohort	South Korea	2002-2012	416,708	50 means	7	Fracture	8
Leslie et al. (2019) (26)	Cohort	Canada	2005-2016	36,996	50 means	7	Osteoporosis, Fracture	7
Stumpf et al. (2020) (16)	Cohort	UK	2003-2018	4,115	69 means	10	Fracture	6

### Risk of bias assessment

The methodological quality of the included studies was assessed using the Newcastle-Ottawa Scale (NOS). The NOS scores ranged from 6 to 9, suggesting moderate to high methodological quality and an overall acceptable risk of bias (**Table 1**).

### Breast cancer and risk of osteoporosis

**Figure 2:**
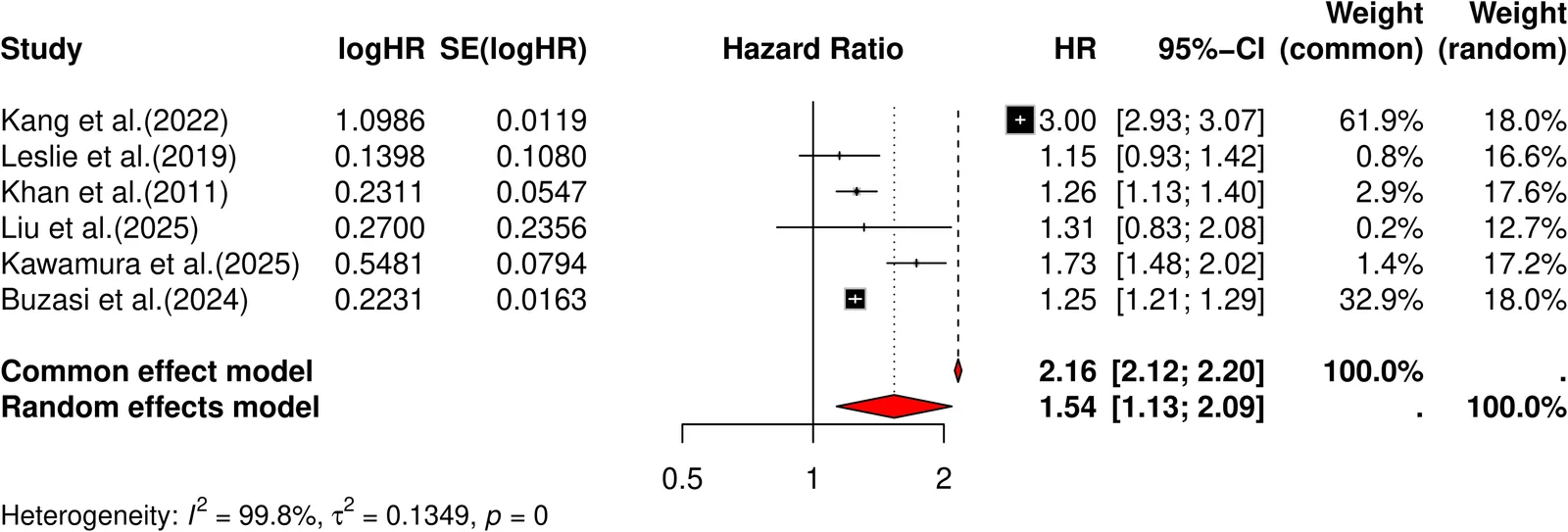
Forest plot showing the association between breast cancer and osteoporosis risk.

Among the 13 studies reporting osteoporosis-related outcomes, six provided overall effect estimates suitable for quantitative synthesis of the association between breast cancer and osteoporosis risk, whereas the remaining studies contributed data only to treatment-specific subgroup analyses. Given the expected clinical and methodological heterogeneity among the included studies, random-effects models were used for all pooled analyses. Substantial statistical heterogeneity was observed across studies (I² = 99.8%, P < 0.001). The pooled analysis demonstrated that breast cancer was associated with a significantly increased risk of osteoporosis, with a combined hazard ratio (HR) of 1.54 (95% CI: 1.13-2.09) (**Figure 2**). These findings indicate that breast cancer patients experience an approximately 54% higher risk of osteoporosis than individuals without breast cancer.

To explore potential sources of heterogeneity, subgroup analyses were conducted according to age and treatment modality. Regarding age, two studies reported osteoporosis risk stratified by age groups. Both studies showed comparable risks of osteoporosis between younger and older breast cancer patients; however, the limited number of studies available for each subgroup precluded further quantitative synthesis.

**Figure 3:**
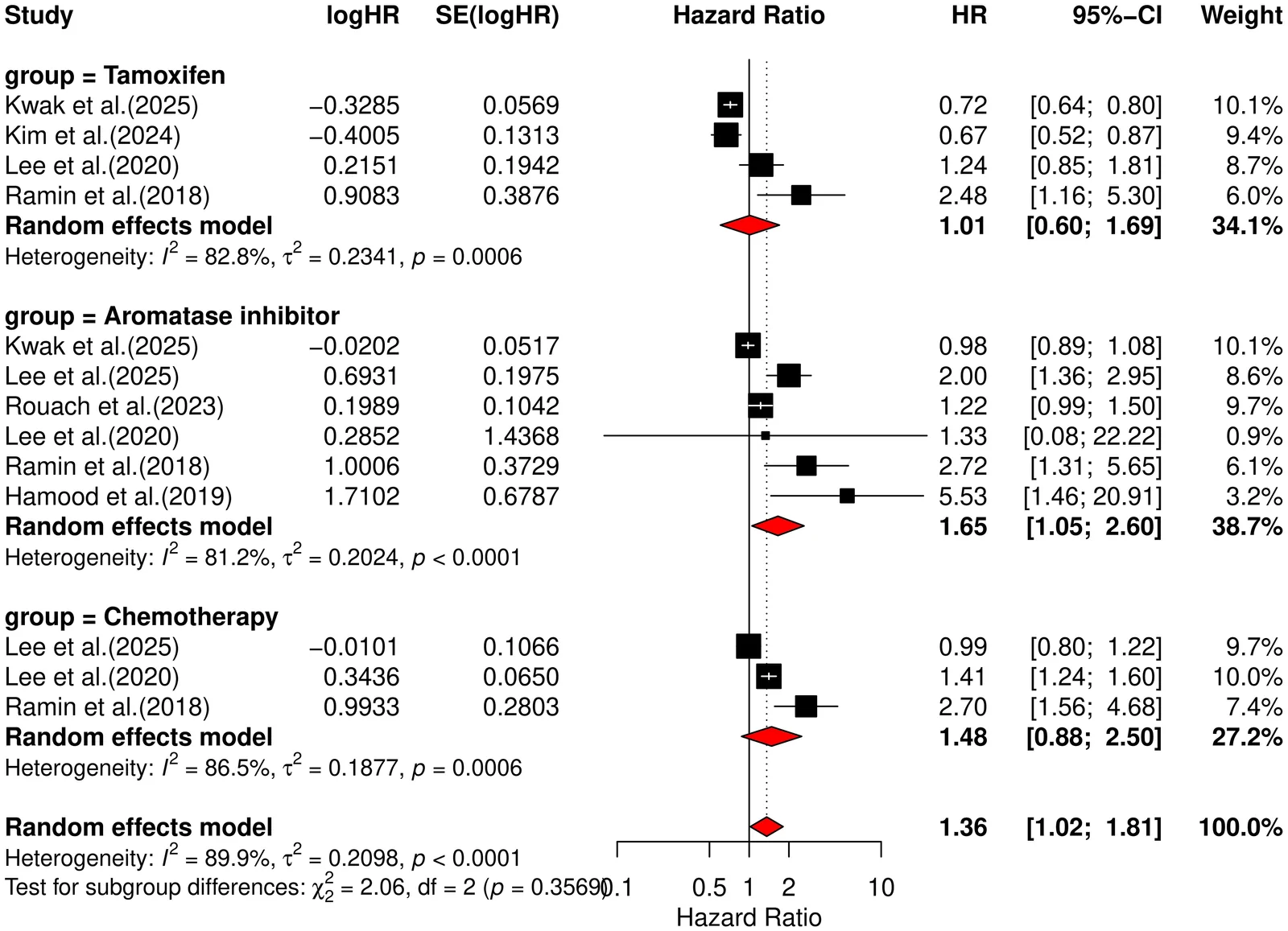
Forest plot of the treatment modality subgroup analysis for the association between breast cancer and osteoporosis risk.

When stratified by treatment modality, a significant association was observed only among patients receiving aromatase inhibitors (HR = 1.65; 95% CI: 1.05-2.60; I² = 81.2%) (**Figure 3**). In contrast, the pooled estimates for tamoxifen users (HR = 1.01; 95% CI: 0.60-1.69; I² = 82.8%) and patients receiving chemotherapy (HR = 1.48; 95% CI: 0.88-2.50; I² = 86.5%) did not reach statistical significance. Nevertheless, no significant difference was detected across treatment subgroups (P = 0.357).

Sensitivity analysis was performed using a leave-one-out approach. Sequential omission of each individual study did not materially alter the pooled effect estimate, with all recalculated HRs remaining statistically significant. These findings indicate that the association between breast cancer and osteoporosis risk was robust and was not driven by any single study (**Supplementary Figure 1**).

Funnel plot inspection did not reveal substantial asymmetry; however, the number of included studies was limited, and therefore the possibility of publication bias could not be completely excluded (**Supplementary Figure 2**).

### Breast cancer and risk of fracture

**Figure 4:**
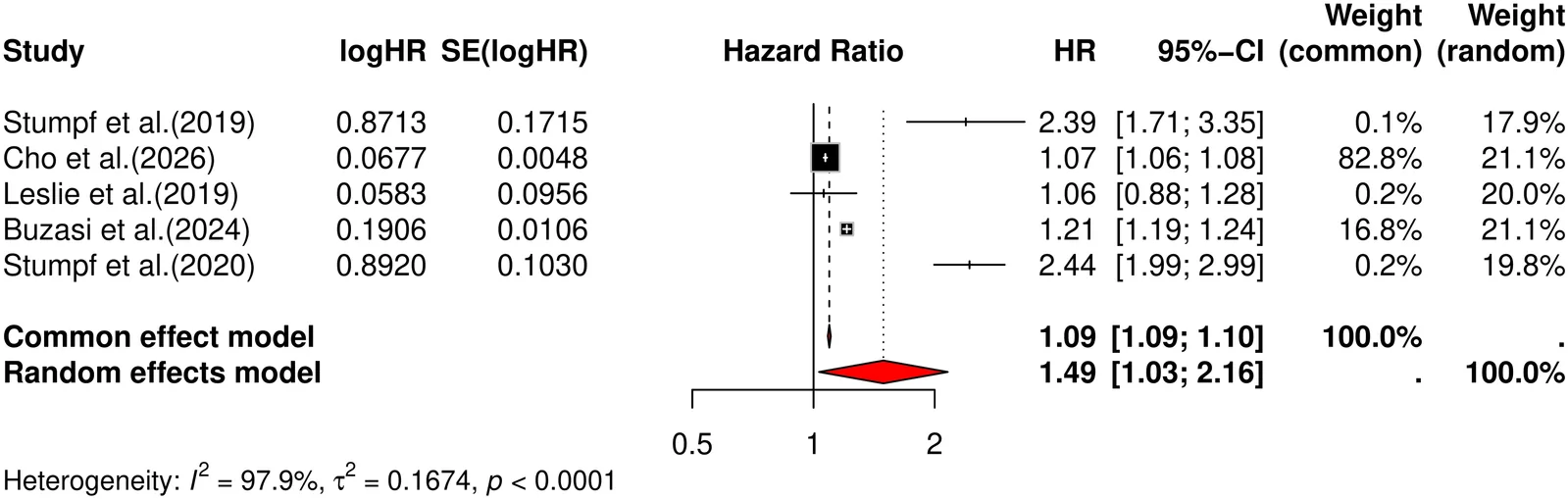
Forest plot showing the association between breast cancer and fracture risk.

Five studies reporting fracture outcomes contributed five effect estimates for quantitative synthesis. Given the high level of heterogeneity among studies (I² = 97.9%, P < 0.001), a random-effects model was adopted. The pooled analysis showed that breast cancer patients had a significantly elevated risk of fracture compared with controls (HR = 1.49; 95% CI: 1.03-2.16) (**Figure 4**), corresponding to an approximately 50% increase in fracture risk.

Leave-one-out sensitivity analysis showed that the pooled effect estimates remained broadly consistent after sequential exclusion of individual studies. Although omission of individual studies slightly attenuated the statistical significance of the association in some analyses, the direction and magnitude of the effect remained largely unchanged, indicating that the overall findings were relatively stable (**Supplementary Figure 3**).

The funnel plot appeared broadly symmetrical, suggesting no obvious evidence of publication bias (**Supplementary Figure 4**). Nevertheless, interpretation should be made with caution because the number of included studies was limited.

## Discussion

In this meta-analysis, we comprehensively evaluated the associations between breast cancer and the risks of osteoporosis and fracture. The pooled results demonstrated that breast cancer patients had significantly increased risks of both osteoporosis (HR = 1.54, 95% CI: 1.13-2.09) and fracture (HR = 1.49, 95% CI: 1.03-2.16). These findings suggest that skeletal complications represent an important long-term health concern among breast cancer survivors.

To explore potential sources of heterogeneity, subgroup analyses were performed according to treatment modality, and age-stratified findings were additionally evaluated. Age-stratified analyses suggested comparable osteoporosis risks between women aged <50 years and those aged ≥50 years; however, the limited number of available studies precluded further quantitative synthesis. In contrast, treatment-specific analyses revealed that only aromatase inhibitor therapy was significantly associated with an increased risk of osteoporosis, whereas no statistically significant associations were observed among tamoxifen users or patients receiving chemotherapy. The lack of a significant association in the tamoxifen subgroup may be attributable to the complex effects of tamoxifen on bone metabolism. Previous studies have shown that tamoxifen may accelerate bone loss in premenopausal women who maintain ovarian function (30), whereas it may attenuate bone loss in women with chemotherapy-induced amenorrhea or postmenopausal status. These opposing effects may have diluted the overall association observed in the present analysis. Although the differences between treatment subgroups did not reach statistical significance, the higher risk observed among aromatase inhibitor users is biologically plausible and consistent with previous clinical evidence (26, 27, 31).

Several mechanisms may explain the increased risks of osteoporosis and fracture observed in breast cancer patients. Estrogen plays a central role in maintaining bone homeostasis by regulating the balance between bone formation and bone resorption (32, 33). Endocrine therapies, particularly aromatase inhibitors, markedly reduce circulating estrogen levels, thereby accelerating bone turnover and bone loss (34, 35). In addition, chemotherapy-induced ovarian failure, chronic inflammation, reduced physical activity, nutritional deficiencies, and vitamin D insufficiency may further contribute to skeletal deterioration (36, 37). As bone mineral density declines, susceptibility to fragility fractures correspondingly increases.

Our findings are supported by previous studies demonstrating that breast cancer survivors, especially those receiving aromatase inhibitor therapy, experience accelerated bone loss and increased fracture risk. Several current clinical guidelines therefore recommend routine bone health assessment, adequate calcium and vitamin D supplementation, and consideration of anti-resorptive therapy in high-risk patients (35). The present study further highlights the importance of early osteoporosis screening and fracture prevention strategies among breast cancer survivors.

This study has several strengths. To our knowledge, it represents an updated meta-analysis evaluating the associations between breast cancer and the risks of osteoporosis and fracture. All included studies were of moderate to high methodological quality according to the Newcastle–Ottawa Scale, and sensitivity analyses generally supported the robustness of the pooled estimates. In addition, this study evaluated both disease-related and treatment-related factors by performing subgroup analyses according to age and treatment modality, providing further insights into the potential factors influencing skeletal outcomes among breast cancer survivors.

Nevertheless, several limitations should be acknowledged. First, substantial heterogeneity was observed across the included studies, particularly in the osteoporosis analysis, and could not be fully explained by the available subgroup analyses. This heterogeneity may be related to differences in population characteristics, geographic regions, treatment regimens, follow-up durations, outcome definitions, data sources, and covariates included in the adjusted models. However, the relatively limited number of studies available for quantitative synthesis restricted further exploration of potential sources of heterogeneity, such as meta-regression analyses. Second, different effect measures, including hazard ratios (HRs), relative risks (RRs), and standardized incidence ratios (SIRs), were pooled in this meta-analysis. Although these measures were used to evaluate the relative association between breast cancer and skeletal outcomes, they represent different statistical quantities and may introduce potential methodological heterogeneity. Therefore, the pooled estimates should be interpreted with caution. Third, several subgroup analyses included a limited number of studies, reducing the statistical power to detect significant subgroup differences. Furthermore, clinically relevant subgroup analyses, such as those based on menopausal status, HER2-targeted therapy, and treatment modality or age among patients with fracture outcomes, could not be performed because of insufficient available data. Fourth, although adjusted estimates were extracted whenever available, residual confounding could not be completely excluded because the included studies were observational in design. Finally, the number of studies available for both osteoporosis and fracture outcomes was relatively small, which limited the statistical power of publication bias assessments. Although funnel plots and Begg’s test did not indicate substantial publication bias, these results should be interpreted cautiously because such methods have limited reliability when the number of included studies is small.

## Conclusion

This meta-analysis suggests that breast cancer is associated with significantly increased risks of both osteoporosis and fracture. Aromatase inhibitor therapy may further contribute to bone loss and skeletal fragility, whereas no statistically significant associations were observed among tamoxifen users or patients receiving chemotherapy. These findings emphasize the importance of bone health monitoring and fracture prevention in breast cancer survivors. Future large-scale prospective studies are warranted to further clarify the effects of different treatment modalities and to identify optimal strategies for preserving skeletal health in this population.
