# Supplementary material for: Risk of osteoporosis and fracture among patients with breast cancer: a systematic review and meta-analysis of cohort studies
Source: Front Oncol. 2026 Aug 31;16:1905334. doi: 10.3389/fonc.2026.1905334 (PMC13572274; doi:10.3389/fonc.2026.1905334)
Supplement: Supplementary file 1 [file DataSheet1.docx]

| **Table S1.** **details of search strategy (from database inception to May 1, 2026)** | | |
| --- | --- | --- |
| **No.** | **Search Strategies** | **Results** |
| **PubMed** | | |
| #1 | "Breast Neoplasms"[Mesh] | 382,772 |
| #2 | (((("Breast Neoplasms"[Mesh]) OR (breast cancer[Title/Abstract])) OR (breast neoplasm[Title/Abstract])) OR (breast tumor[Title/Abstract])) OR (breast carcinoma[Title/Abstract]) | 520,052 |
| #3 | "Osteoporosis"[Mesh] | 69,755 |
| #4 | "Fractures, Bone"[Mesh] | 227,448 |
| #5 | (((((osteoporosis[Title/Abstract]) OR (osteopenia[Title/Abstract])) OR (bone loss[Title/Abstract])) OR (fracture[Title/Abstract])) OR (bone fracture[Title/Abstract])) OR (fragility fracture[Title/Abstract]) | 465,767 |
| #6 | #2 AND (#3 OR #4 OR #5) | 5,161 |
| #7 | ((("Cohort Studies"[Mesh]) OR (cohort[Title/Abstract])) OR (case-control[Title/Abstract])) OR (case control[Title/Abstract]) | 3,492,928 |
| #8 | #6 AND #7 | 871 |
| **Embase** | | |
| #1 | ‘breast cancer’/exp | 845,276 |
| #2 | 'breast cancer':ab,ti OR 'breast neoplasm':ab,ti OR 'breast tumor':ab,ti OR 'breast carcinoma':ab,ti | 629,142 |
| #3 | ‘osteoporosis’/exp | 186,760 |
| #4 | ‘bone fracture’/exp | 458,752 |
| #5 | 'osteoporosis':ab,ti OR 'osteopenia':ab,ti OR 'bone loss':ab,ti OR 'fracture':ab,ti OR 'bone fracture':ab,ti OR 'fragility fracture':ab,ti | 453,333 |
| #6 | (#1 OR #2) AND (#3 OR #4 OR #5) | 15,137 |
| #7 | ‘cohort studies’/exp | 1,689,413 |
| #8 | 'cohort':ab,ti OR 'case-control':ab,ti OR 'case control':ab,ti | 2,056,985 |
| #9 | #6 AND (#7 OR #8) | 1,355 |
| **Cochrane Library** | | |
| #1 | MeSH descriptor: [Breast Neoplasms] explode all trees | 25,376 |
| #2 | (breast cancer):ab,ti,kw OR (breast neoplasm):ab,ti,kw OR (breast tumor):ab,ti,kw OR (breast carcinoma):ab,ti,kw | 53,226 |
| #3 | MeSH descriptor: [Osteoporosis] explode all trees | 14,679 |
| #4 | MeSH descriptor: [Fractures, Bone] explode all trees | 12,009 |
| #5 | (osteoporosis):ab,ti,kw OR (osteopenia):ab,ti,kw OR (bone loss):ab,ti,kw OR (fracture):ab,ti,kw OR (bone fracture):ab,ti,kw OR (fragility fracture):ab,ti,kw | 44,965 |
| #6 | MeSH descriptor: [Cohort Studies] explode all trees | 42,247 |
| #7 | (cohort):ab,ti,kw OR (case-control):ab,ti,kw OR (case control):ab,ti,kw | 126,419 |
| #8 | (#1 OR #2) AND (#3 OR #4 OR #5) AND (#6 OR #7) | 139 |
| **Web of science** | | |
| #1 | Breast Neoplasms OR breast cancer OR breast neoplasm OR breast tumor OR breast carcinoma | 877,359 |
| #2 | Osteoporosis OR osteopenia OR bone loss OR fracture OR bone fracture OR fragility fracture | 962,800 |
| #3 | #1 AND #2 | 9,463 |
| #4 | Cohort Studies OR cohort OR case-control OR case control | 2,457,000 |
| #5 | #3 AND #4 | 1,058 |

| **Table S2. Reason description for exclusion of 29 studies** | | | |
| --- | --- | --- | --- |
| **No.** | **First author** | **Publication year** | **Reason for exclusion** |
| 1 | Evelyn H | 2018 | Case-control study |
| 2 | Shuman Y | 2022 | Case-control study |
| 3 | Hyo Geun C | 2021 | Lack of relevant data |
| 4 | Brendan L M | 2021 | Lack of relevant data |
| 5 | David A | 2024 | Lack of relevant data |
| 6 | Deborah C | 2024 | Lack of relevant data |
| 7 | Alisa D K | 2007 | Meta-analyses |
| 8 | B F | 2014 | Meta-analyses |
| 9 | Yingfang D | 2018 | Meta-analyses |
| 10 | Christian Tang A | 2018 | No outcome of interest |
| 11 | Wolfgang B | 2018 | No outcome of interest |
| 12 | R H | 2019 | No outcome of interest |
| 13 | Joan C L | 2021 | No outcome of interest |
| 14 | Naoko T | 2022 | No outcome of interest |
| 15 | Yanyu Z | 2022 | No outcome of interest |
| 16 | Gherardo M | 2022 | No outcome of interest |
| 17 | Alessandro d S | 2022 | No outcome of interest |
| 18 | Irene R | 2022 | No outcome of interest |
| 19 | Yadav N | 2023 | No outcome of interest |
| 20 | Carl B | 2024 | No outcome of interest |
| 21 | Nicola L B | 2024 | No outcome of interest |
| 22 | Ichiro I | 2025 | No outcome of interest |
| 23 | Sabin Goktas A | 2025 | No outcome of interest |
| 24 | Rebecca P | 2026 | No outcome of interest |
| 25 | B J E | 2016 | No full text |
| 26 | David H | 2018 | No full text |
| 27 | Soo Hyun K | 2019 | No full text |
| 28 | Tanya G | 2022 | No full text |
| 29 | Emily L | 2024 | No full text |
| **Excluded studies for the table[1-29]**  1. Hsieh, E., et al., *Vertebral fractures among breast cancer survivors in China: a cross-sectional study of prevalence and health services gaps.* BMC Cancer, 2018. **18**(1): p. 104.  2. Yang, S., et al., *Association between prior cancer diagnosis and osteoporosis: a matched case-control study.* Arch Osteoporos, 2022. **17**(1): p. 112.  3. Choi, H.G., et al., *Analyses of the association between breast cancer and osteoporosis/fracture history: a cross-sectional study using KoGES HEXA data.* Arch Osteoporos, 2021. **16**(1): p. 98.  4. McNeish, B.L., et al., *Chemotherapy-induced peripheral neuropathy increases nontraumatic fracture risk in breast cancer survivors.* JBMR Plus, 2021. **5**(8): p. e10519.  5. Abbad-Gomez, D., et al., *Effect of comorbidity and multimorbidity on adherence to follow-up recommendations among long-term breast cancer survivors.* Maturitas, 2024. **182**: p. 107918.  6. Cosentini, D., et al., *Fat Body Mass and Vertebral Fracture Progression in Women With Breast Cancer.* JAMA Netw Open, 2024. **7**(1): p. e2350950.  7. Kjaergaard, A.D., et al., *Estrogen receptor alpha polymorphism and risk of cardiovascular disease, cancer, and hip fracture: cross-sectional, cohort, and case-control studies and a meta-analysis.* Circulation, 2007. **115**(7): p. 861-71.  8. Frediani, B., E. Baraldi, and G. Cremonesi, *Effect of clodronate treatment on risk of fracture: a systematic review and meta-analysis.* Calcif Tissue Int, 2014. **95**(4): p. 295-307.  9. Deng, Y., et al., *Oral bisphosphonates and incidence of cancers in patients with osteoporosis: a systematic review and meta-analysis.* Arch Osteoporos, 2018. **14**(1): p. 1.  10. Axelsen, C.T., et al., *Bone loss during neoadjuvant/adjuvant chemotherapy for early stage breast cancer: A retrospective cohort study.* Mol Clin Oncol, 2018. **8**(6): p. 767-772.  11. Brozek, W., et al., *Bone Mineral Density and Breast Cancer Incidence and Mortality in Postmenopausal Women: A Long-Term Follow-Up Study.* J Womens Health (Larchmt), 2019. **28**(5): p. 628-635.  12. Hamood, R., et al., *Hormone therapy and osteoporosis in breast cancer survivors: assessment of risk and adherence to screening recommendations.* Osteoporos Int, 2019. **30**(1): p. 187-200.  13. Lo, J.C., et al., *Description of Major Osteoporotic Fractures in Women with Invasive Breast Cancer Who Received Endocrine Therapy.* JAMA Netw Open, 2021. **4**(11): p. e2133861.  14. Tanda, N., et al., *Influence of alcohol sensitivity on bone metastases and skeletal-related events in primary operable breast cancer: A retrospective cohort study.* PLoS One, 2022. **17**(6): p. e0269335.  15. Zhang, Y., et al., *Bone mineral density and risk of breast cancer: A cohort study and Mendelian randomization analysis.* Cancer, 2022. **128**(14): p. 2768-2776.  16. Mazziotti, G., et al., *Real-World Effectiveness of Denosumab and Bisphosphonates on Risk of Vertebral Fractures in Women with Breast Cancer Undergoing Treatment with Aromatase Inhibitors.* Calcif Tissue Int, 2022. **111**(5): p. 466-474.  17. de Sire, A., et al., *Vitamin D Deficiency in Women with Breast Cancer: A Correlation with Osteoporosis? A Machine Learning Approach with Multiple Factor Analysis.* Nutrients, 2022. **14**(8).  18. Rodríguez-Gómez, I., et al., *Osteoporosis and Its Association With Cardiovascular Disease, Respiratory Disease, and Cancer: Findings From the UK Biobank Prospective Cohort Study.* Mayo Clin Proc, 2022. **97**(1): p. 110-121.  19. Nisha, Y., et al., *Cytotoxic chemotherapy is associated with decreased bone mineral density in postmenopausal women with early and locally advanced breast cancer.* Arch Osteoporos, 2023. **18**(1): p. 41.  20. Blomqvist, C., et al., *Long-term effects of aromatase inhibitor withdrawal on bone mineral density in early breast cancer patients: 10-year follow-up results of the BREX study.* Breast Cancer Res Treat, 2024. **206**(1): p. 57-65.  21. Barclay, N.L., et al., *Collateral effects of the COVID-19 pandemic on endocrine treatments for breast and prostate cancer in the UK: a cohort study.* Ther Adv Med Oncol, 2024. **16**: p. 17588359241253115.  22. Itonaga, I., et al., *Prosthetic joint replacement for femoral bone metastases and pathological fracture: A retrospective case series study of cases within the past 10 years.* Medicine (Baltimore), 2025. **104**(52): p. e46927.  23. Goktas Aydin, S., et al., *A multicenter prospective matched comparative study on osteoporosis risk in cancer patients receiving chemotherapy.* Support Care Cancer, 2025. **33**(12): p. 1053.  24. Pedersini, R., et al., *Fracture risk in metastatic breast cancer patients treated with CDK 4/6 inhibitors and endocrine therapy.* Breast Cancer Res Treat, 2026. **215**(2): p. 58.  25. Edwards, B.J., et al., *Elevated incidence of fractures in women with invasive breast cancer.* Osteoporos Int, 2016. **27**(2): p. 499-507.  26. Henault, D., et al., *Divergence from osteoporosis screening guidelines in older breast cancer patients treated with anti-estrogen therapy: A population-based cohort study.* Bone, 2018. **116**: p. 94-102.  27. Kim, S.H., et al., *Changes in Bone Mineral Density in Women With Breast Cancer: A Prospective Cohort Study.* Cancer Nurs, 2019. **42**(2): p. 164-172.  28. Gupta, T., et al., *Incident comorbidities after tamoxifen or aromatase inhibitor therapy in a racially and ethnically diverse cohort of women with breast cancer.* Breast Cancer Res Treat, 2022. **196**(1): p. 175-183.  29. Liang, E., et al., *A prospective study of vitamin D, proinflammatory cytokines, and risk of fragility fractures in women on aromatase inhibitors for breast cancer.* Breast Cancer Res Treat, 2024. **208**(2): p. 349-358. | | | |

**Supplementary Figure S1**. Sensitivity analysis for the association between breast cancer and osteoporosis risk.

**Supplementary Figure S2**. Funnel plot for publication bias assessment of studies investigating the association between breast cancer and osteoporosis risk.

**Supplementary Figure S3**. Sensitivity analysis for the association between breast cancer and fracture risk.

**Supplementary Figure S4**. Funnel plot for publication bias assessment of studies investigating the association between breast cancer and fracture risk.
